# Association between utilisation of pre-hospital emergency services and premature discharge in Emergency Department attendances with mental health triage codes: a retrospective cohort study

**DOI:** 10.1186/s12873-026-01638-0

**Published:** 2026-06-16

**Authors:** R. Moola, R. M. Duffy, A Moughty, A. M. Doherty

**Affiliations:** 1 Department of Liaison Psychiatry, Mater Misericordiae University Hospital, Dublin, Ireland; 2 Department of Psychiatry, University College Dublin, Dublin, Ireland; 3 Department of Emergency Medicine, Mater Misericordiae University Hospital, Dublin, Ireland

**Keywords:** Self-injurious behaviour, Triage, Mental health service, Emergency services, Psychiatric, Emergency medicine

## Abstract

**Background:**

People attending the Emergency Department (ED) with mental health conditions are a vulnerable group who may struggle to engage with scheduled care. Existing research suggests this cohort are more likely to leave before treatment is complete. This study aimed to examine the utilisation rates of pre-hospital emergency services in transferring people with mental health triage codes to the ED, establish rates on incomplete care, and examine any associations between mode of arrival and self-discharge prior to completion of care.

**Methods:**

This study utilised retrospective analysis of attendances at an inner-city ED on individuals presenting with a mental health triage code: mental illness; behaving strangely; overdose & poisoning; and self-harm. SPSS was used to perform descriptive, bivariate, and multivariate analyses of patient characteristics and the associations between mode of arrival and self-discharge from 2008 to 2022.

**Results:**

During the study period 30,941 patients presented to the ED with mental health triage codes. Of these, 53% (*n* = 16,390) were brought by ambulance and 3.3% (*n* = 1,016) by the police service. In total, 28.4% (*n* = 8,732) left before completion of care. Self-discharge was significantly associated with arrival by ambulance (*p* < 0.001), less urgent triage categories (*p* < 0.001), and male gender (*p* < 0.001). The association between self-discharge and mode of arrival remained significant after controlling for age and gender, triage code and time of arrival (OR = 1.42; *p* < 0.001).

**Conclusion:**

In people attending the ED with mental health problems, self-discharge is more frequent in those brought by ambulance. There is a need for qualitative data to understand the needs of this population and target interventions to avoid premature cessation of treatment.

**Clinical trial number:**

Not applicable.

## Background

People with mental illness presenting to the Emergency Department (ED) are a vulnerable group, and many struggle to engage with scheduled care in the community. These individuals often have multiple risk factors which impact the delivery of healthcare, including physical co-morbidities, substance misuse, homelessness, and other social issues. These factors can impact health, help seeking behaviours, and access to healthcare. As a result, there may be an overreliance on unscheduled rather than scheduled care. For many people, their first engagement with mental health services occurs via the ED, and those with established mental illness present with both emergency and non-emergency concerns [1]. The ED, along with liaison psychiatry services based there, provide a critical role in ensuring those with mental illness access the care they require, and may be the only or most accessible option available to some.

Mental health indications comprise a significant amount of all ED presentations. In Australia, 3% of all public ED presentations between 2021 and 2022 were mental health related [2]. English National Health Service (NHS) data shows a greater than 200% increase in ED presentations for all mental health indications between 2010 and 2022 [3, 4]. In Ireland, while we lack data on all-cause mental health ED presentations, there were 14,555 self-harm presentations to all EDs in the two years 2017–2018 [5].

Existing literature suggests that people with mental illness are more likely to be brought to ED by ambulance or the police than the general public [6]. Data from the Australian Institute of Health and Welfare in 2022 reported that those with mental illness arrived by ambulance or helicopter in 50% of cases, with 8% being transported by police or correctional service vehicles, and 42% self-presenting [2]. This is in comparison to overall population figures where 26% arrived by ambulance, 1% by police, and 74% self-presented. Mental health care creates a sizeable caseload for ambulance and police services who are frequently under-resourced to adequately service this cohort. In the UK, it is estimated that 15% of all police callouts involve people with a mental health issue. This has raised concerns, from both the public and police, with suggestions that police forces are insufficiently trained to provide initial mental health care [7].

Not all patients presenting to the ED wait to be seen, and for various reasons may leave before care is complete. Many EDs lack the resources and specialised psychiatric services required to meet the needs of those in crisis. A limited availability of psychiatric beds can result in prolonged waiting times [8]. This population is twice as likely to spend 12 h or more in ED [9], while the wait for an inpatient bed or facility transfer can be up to three times longer [10].

Among those treated for self-harm in English hospitals, alcohol or illicit drug use increased the risk of incomplete care by 49% [11]. This is of concern as rates of completed suicide are up to 30-times greater for those attending ED with self-harm [12–14]. In qualitative studies, stigma from ED staff has been reported by those in crisis, increasing desire to leave [15, 16].

Understanding how people access care and understanding risk factors associated with incomplete care may help focus mental healthcare delivered in the ED. Understanding the needs of this cohort can inform the development of alternative models of care which better address mental health specific needs.

The study aimed to examine the utilisation rates of pre-hospital emergency services in transferring people with mental health triage codes to the ED, establish rates on incomplete care, and examine any associations between mode of arrival and self-discharge prior to completion of care.

## Methods

### Design

This is a retrospective cohort study using clinical registry data from a major inner-city teaching hospital in Dublin, Ireland. The hospital’s Electronic Patient Records (EPR) was used to identify all patients who presented to the ED and were triaged with a mental health triage code between 2008 and 2022 (a 15-year cohort).

### Study setting

This study was conducted at the ED of a tertiary centre which provides multiple national and regional specialist services. The local catchment area is approximately 185,000 people, with over 89,000 patients attending the ED in 2021. Patients requiring an emergency mental health assessment can self-present to ED or be transferred by ambulance or police. The majority of those recommended for admission from the community under the Mental Health Act 2001 bypass ED assessment and are brought directly to an approved centre (psychiatric unit). They should only be transferred to the ED while under the Mental Health Act if a physical condition necessitates emergency treatment. Prior to January 2021, there was one local alternative 24/7 site where people in mental health crisis could access unscheduled care. This closed due to staffing and lack of onsite medical access for those with co-morbid physical health needs. Since closure, the ED provides the only local access to emergency mental healthcare. Parallel assessment was introduced in 2020: this is considered best practice and involves commencing mental health assessment before the patients’ medical care is complete, or in parallel with medical care [17, 18]. This avoids unnecessary delays for patients to be “medically cleared” prior to psychiatric review.

### Sample

This study examined the register of attendances between 2008 and 2022 to the Mater Misericordiae University Hospital ED. At nursing triage, presumed mental health presentations were assigned one or more of four triage codes following brief nursing assessment as per normal protocol: mental illness; behaving strangely; overdose and poisoning; or self-harm. The sample included all patients presenting to triage assigned one of the above triage codes. Further clinical variables were selected to capture in detail the patient’s journey through the ED.

The following data were extracted from the EPR for all people who presented with a mental health triage code:

1. Sociodemographic factors including age, sex, area of residence.
2. Clinical variables including:
  - Presenting problem.
  - Mode of arrival – self, ambulance, police.
  - Self-harm – means, treatment required.
  - Admission to hospital – psychiatric admission or medical/surgical admission.
  - Medical treatment required – ED assessment/ medical team review/ medical admission/ critical care admission.
  - Left before treatment complete.
  - Time and day of arrival to ED.
  - Waiting time in the ED.
  - Triage category.
3. Service use.
  - Number of ED attendances in the previous year.

### Data analysis

Data were extracted as an Excel file and entered into SPSS. Utilising SPSS, descriptive and bivariate analyses of patient presentations with these triage codes were performed. The associations between mode of arrival and incomplete care were explored utilising chi-squared tests and t-tests. Multivariable analysis was performed utilising logistic regression with leaving before completion of treatment as the dependent variable. The significance level was set at 0.05.

### Ethics

This study was conducted in accordance with the guidelines of the World Medical Association’s Declaration of Helsinki [19]. Ethical approval was granted by the Mater Misericordiae University Hospital’s Research Ethics Committee (1/378/2327TMR).

## Results

Over the 15-year period studied, 30,941 patients presented to the ED with mental health triage codes. Three cases with incomplete data were excluded, analysing the remaining 30,938. Table 1 describes characteristics of the cohort.

**Table 1:** Characteristics of people (*n* = 30,938) presenting to the Emergency Department with Mental Health Triage Codes 2008–2022

	Total (*n* = 30938)	Planned discharge from ED (*n* = 22146) *n* (%)	Discharge before completion of care (*n* = 8792) *n* (%)	Chi Sq (*p*-value)
Gender:				
Male	18,173 (58.7)	12,541 (56.6)	5632 (64.1)	145.1 (< 0.001)
Female	12,759 (41.2)	9599 (43.4)	3160 (35.9)	
Other	6 (0)	6 (0)	0 (0)	
Triage category:				
Immediate Very urgent Urgent Standard	264 (0.9) 18,634 (60.2) 11,650 (37.7) 390 (1.3)	203 (0.9) 14,812 (66.9) 6955 (31.4) 176 (0.8)	61 (0.7) 3822 (43.5) 4695 (53.4) 214 (2.4)	1519.2 (< 0.001)
Mode of arrival (*n* = 30937):				
Self Ambulance Gardaí	13,532 (43.7) 16,389 (53) 1016 (3.3)	10,176 (46) 11,153 (50.4) 816 (3.7)	3356 (38.2) 5236 (59.6) 200 (2.2)	225.5 (< 0.001)
Time of arrival:				
8am-8pm 8pm-midnight Midnight − 8am	16,099 (52) 6881 (22.2) 7958 (25.7)	12,141 (54.8) 4753 (21.5) 5252 (23.7)	3958 (45) 2128 (24.2) 2906 (30.8)	259.6 (< 0.001)
Wait time in ED				
< 6 h 6–9 h 9–12 h 12–24 h 24 h+	11,824 (38.2) 6495 (21) 4221 (13.6) 6272 (20.3) 2126 (6.9)	7117 (32.1) 4737 (21.4) 3161 (14.3) 5174 (23.4) 1957 (8.8)	4707 (53.5) 1758 (20) 1060 (12.1) 1098 (12.5) 169 (1.9)	1587.6 (< 0.001)
Triage code:				
Self-Harm Mental Illness Behaving Strangely Overdose & Poisoning	2265 (7.3) 14,613 (47.2) 3370 (10.9) 10,690 (34.6)	1651 (7.5) 10,942 (49.4) 2449 (11.1) 7104 (32.1)	614 (7) 3671 (41.8) 921 (10.5) 3586 (40.8)	220.1 (< 0.001)
Admitting specialty				
Psychiatry Medicine Surgery Emergency Medicine	1734 (36.3) 2586 (54.2) 136 (2.9) 315 (6.6)	1698 (38.5) 2324 (52.7) 129 (2.9) 260 (5.9)	36 (10) 262 (72.8) 7 (1.9) 55 (15.3)	166.3 (< 0.001)
Age:				
Mean (SD) Median (range)	35.97 (SD 13.2) 34 (14–102)	36.4 (13.85) 34 (14–94)	35.99 (11.4) 33 (16–102)	U = 90963090.5 (< 0.001) *r* = 0.025*

**Fig. 1:**
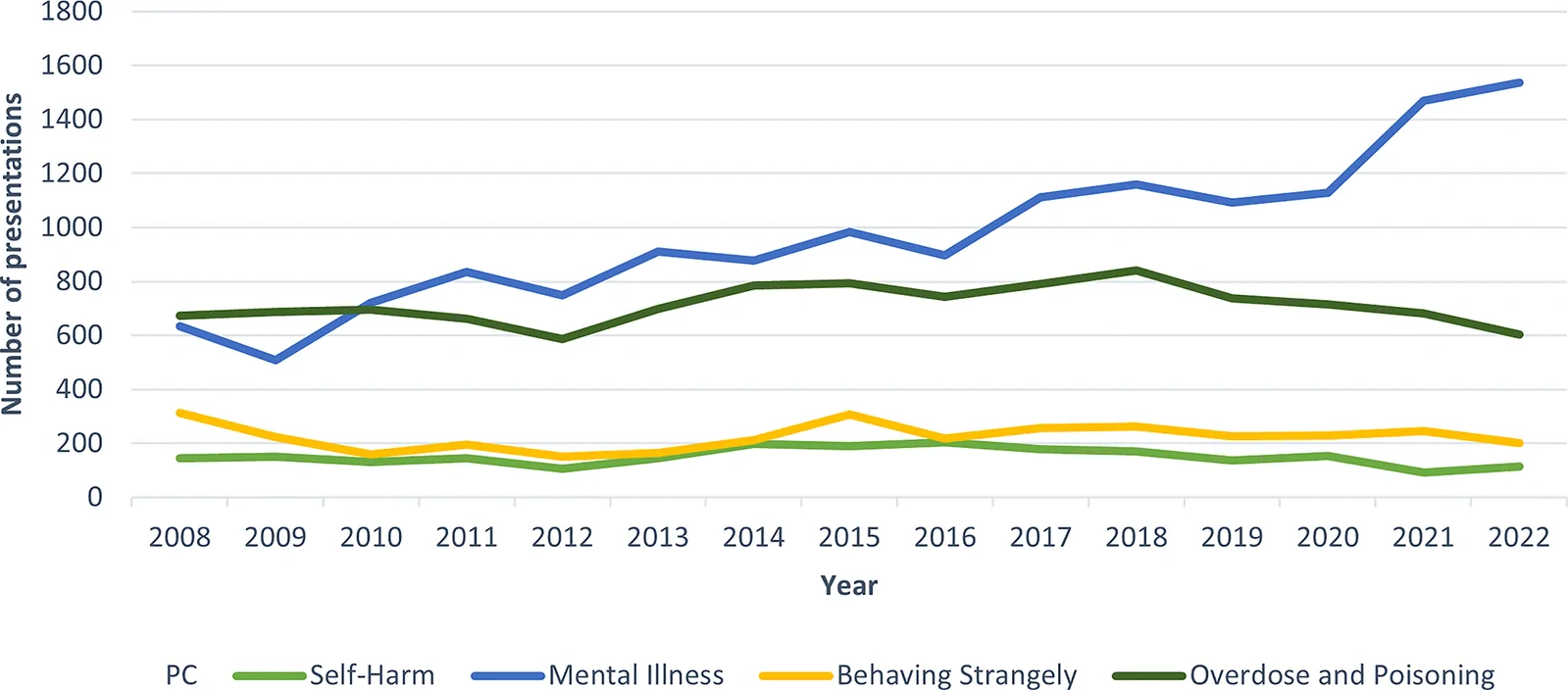
Trends in mental health presentations to the Emergency Department 2008 to 2022, including triage code detail

Trends over time demonstrated a significant increase in individuals presenting with the code ‘mental illness’ (Fig. 1). Of the 30,938 patients studied, 52.9% (n = 16,368) did not require admission and were formally discharged home, 11.4% (n = 3518) were admitted, and 7.3% (n = 2244) transferred to other inpatient psychiatric facilities following evaluation.

**Fig. 2:**
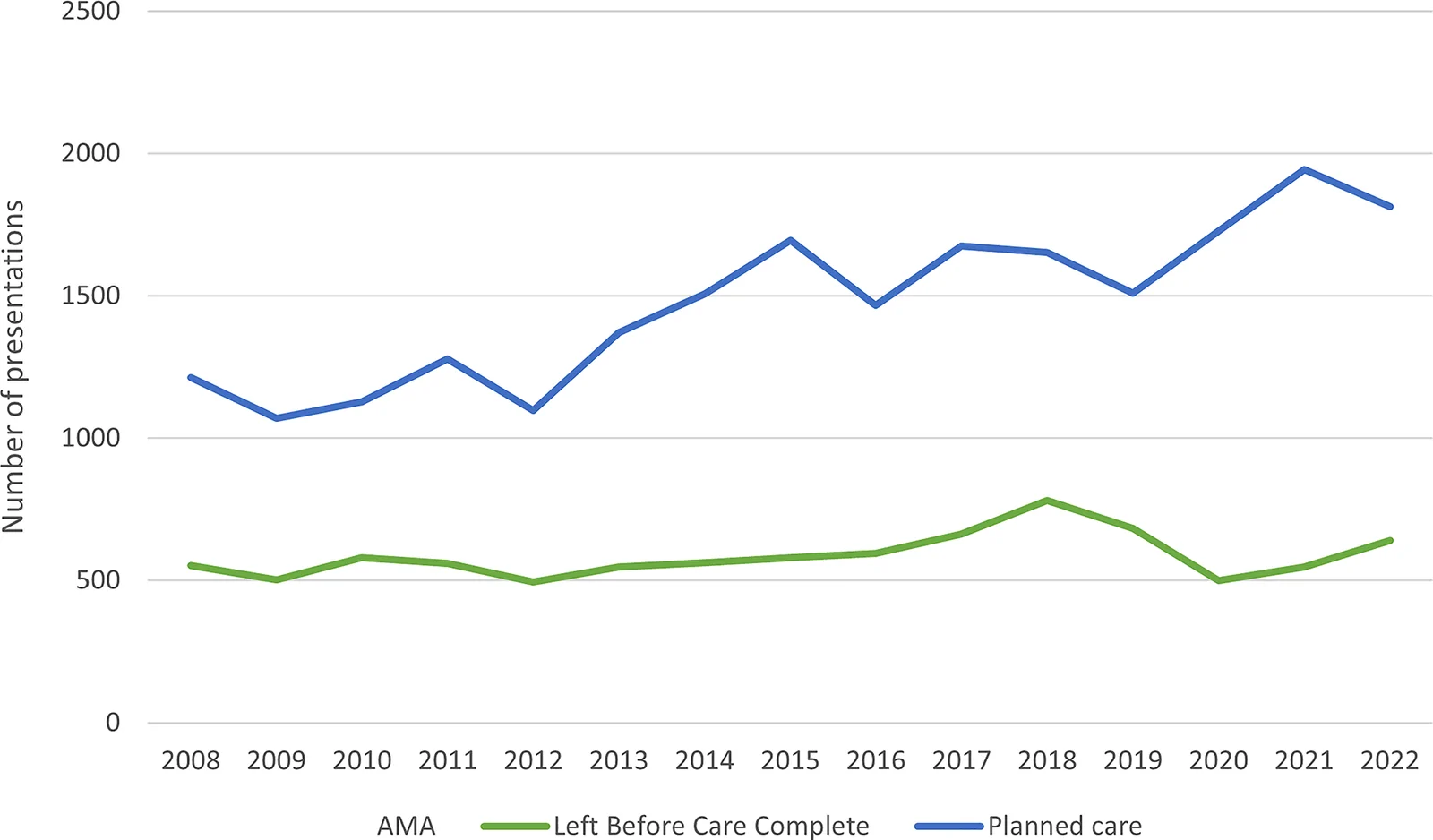
Trends in patients leaving the Emergency Department before care is complete, 2008–2022

One-quarter (28.4%; *n* = 8,792) of individuals left before completion of care. Figure 2 describes the pattern of leaving before completion of care in the study period.

Differences were observed in the rates of people leaving before completion of care across triage codes. One-quarter of people presenting with the code ‘mental illness’ (25.1%; n = 3,671), 27.1% (n = 614) presenting with ‘self-harm’, 27.3% (n = 921) coded as ‘behaving strangely’ and 33.5% (n = 3586) presenting with ‘overdose and poisoning’ left before completion of care. Male patients were significantly more likely to self-discharge (n = 5632, 64.1%: p < 0.001).

As outlined in Table 1, when the mode of arrival of patients who self-discharged were compared with those completing care, individuals transferred to ED by ambulance were significantly more likely to self-discharge (*p* < 0.001). Of those transferred by ambulance, 31.9% (*n* = 5,236) left before completion of care, higher than the proportion among those brought by police (*n* = 200; 19.7%) or who self-presented (*n* = 3356; 24.8%).

There was a significant association between less urgent triage categories and self-discharge. Of the standard triage categories (‘Immediate’, ‘Very Urgent’, ‘Urgent’, ‘Standard’), those in the ‘Very Urgent’ (*n* = 3822; 43.5%) and ‘Urgent’ categories (*n* = 4695; 53.4%) had significantly higher rates of discharge before completion of care (*p* < 0.001). This is in comparison to only 0.7% of patients in the ‘Immediate’ category (*n* = 61) and 2.4% in the ‘Standard’ category (*n* = 214).

People who presented with a mental health problem and left before completion of care had been seen more promptly: they were more frequently seen within 6 h of arrival in ED (*n* = 4707, 53.5%), compared with 32.1% (*n* = 7117) of those who remained until completion of care. There were significantly lower proportions of those who left before care was complete among those who waited more than 24 h to be seen: 1.9% compared with 8.8%.

Using multivariate analysis, the association between self-discharge and mode of arrival remained significant after controlling for age, gender, triage code and time of attendance (*p* < 0.001; CI 0.65–0.897) as described in Table 2. Patients who arrived by ambulance were 1.42 times more likely to leave before completion of care than those who self-presented or arrived accompanied by police (OR 1.42; *p* < 0.001).

**Table 2:** The association between self-discharge and mode of arrival after controlling for age and gender using logistic regression

	B	*p*	Odds Ratio	CI
Age	0.007	< 0.001	1.009	1.007–1.011
Gender	0.317	< 0.001	1.37	1.303–1.446
Mode: Self		< 0.001		
Ambulance	-0.199	< 0.001	1.42	0.775–0.867
Gardaí	0.365	< 0.001	1.34	1.225–1.693
Triage code: Self-Harm		< 0.001		
Mental Illness	-0.006	0.904	1.01	0.897–1.101
Behaving strangely	-0.079	0.208	1.08	0.818–1.045
Overdose & poisoning	-0.289	< 0.001	1.34	0.675–0.83
Time of attendance: 8am-8pm		< 0.001		
8pm-midnight	-0.286	< 0.001	1.33	0.705–0.801
Midnight − 8am	0.431	< 0.001	1.54	0.611–0.69

Of note, those who arrived accompanied by police were 1.34 times more likely to complete care (OR 1.34; *p* < 0.001) than those who arrived by ambulance or self-presented. In addition, the triage codes of ‘overdose and poisoning’ were significantly more likely than all other triage codes to leave prematurely, and those coded as ‘self-harm’ were more likely to stay (*p* < 0.001). Patients presenting between 8pm and midnight were also more likely to leave before completion of care, and those arriving between midnight and 8 am were most likely to receive complete care (*p* < 0.001).

## Discussion

This study described the characteristics associated with self-discharge prior to completion of care for patients arriving at ED with mental health indications. Over a 15-year study period, 28.4% (*n* = 8,792) of patients left ED prior to completion of care. The characteristics of those more likely to leave before care was completed include male sex, arrival by ambulance, younger age, triaged with ‘overdose and poisoning’, and attending late evening/night. Those who arrived accompanied by police were more likely to complete care.

Of note, there was a significant increase in the overall number of people attending the ED with mental health triage codes, and particularly those coded with ‘mental illness’ over the period studied. A similar trend has been noted in the US [20] and has been explored with reference to the COVID-19 pandemic in a range of countries [21, 22]. Changes in local service delivery likely contributed to an increase in presentations with ‘mental illness’ in particular (this category may represent patients suitable for assessment in a location other than ED). Given that this data found a significant increase in 2021 rather than 2020, this likely reflects a key service change in January 2021 resulting in no crisis alternatives to the ED. It is noteworthy that this was not associated with an increase in patients leaving before care was completed: this may be related to the process of parallel assessment, implemented in 2020.

The proportion of 28.4% of people leaving before completion of care is very high by international standards. A study of patients with mental illness in the EDs of New South Wales, Australia noted that 3.13% left before completion of care [23]. A retrospective cohort study of presentations to EDs in Queensland, Australia from 2012 to 2017 reported that 2.8% left before completion of care, with 1.1% of patients leaving prior to engaging in any care [24]. The only similar work conducted in Ireland, from the National Self-Harm Registry 2017 to 2018, reported that 12.1% of people presenting with self-harm left prior to completion of care [5]. This is less than half the rate reported among those with self-harm in this study (27.1%). One South Korean study reported higher rates of self-discharge in a cohort of over 6,000 adolescents where 22.8% left before completion of care [25]. However, this was associated with female gender and older age, unlike in this study where younger age and male gender were more strongly associated with self-discharge.

Regarding the high self-discharge rate amongst those arriving by ambulance, there are likely several contributing factors. For those experiencing an acute mental health crisis, a busy environment with prolonged waiting times can be challenging [26]. For patients arriving by ambulance or the police service, their ED journey may have been initiated by a third party, such as a concerned family member or bystander. Individuals accompanied by police are frequently commenced on security special observations which may prevent departure and may explain the lower proportion of patients in this category leaving prematurely. This is less frequently the case for those arriving by ambulance, and the patient may themselves deprioritise the importance of completion of care. Of note, the proportion of 31.9% of people brought by ambulance who left before completion of care is also high by international standards. A study of Scottish Ambulance Service patient records from 2011 noted that 11% of ambulance callouts involved diagnostic codes relating to mental health emergencies or self-harm [6]. In this cohort, 17% of ED transfers resulted in self-discharge, and those who self-discharged following ambulance transfer were 25% more likely to contact ambulance services for a mental health emergency within the year. Parallel assessment may be beneficial to ensure appropriate provision of care to this vulnerable cohort by ensuring their medical and psychiatric needs are both addressed without delay, de-emphasising the idea that a patient should be “medically cleared” prior to psychiatric input.

The role of alcohol and drugs may contribute to the higher rate of self-discharge, especially among those triaged as ‘overdose and poisoning’. Individuals may leave due to the resolution of a crisis precipitated by intoxication or due to withdrawal symptoms which may not be identified or managed in ED [27]. Those who are significantly intoxicated, along with those presenting with non-urgent care needs may be deprioritised at triage, leading to longer median wait times, and a higher degree of self-discharge.

Targeted interventions to reduce rates of incomplete care by identifying and prioritising those most at risk of self-discharge would be beneficial. The use of peer recovery coaches [28] and referral to rapid-access addiction services [29] have shown promise in patient engagement in ED for those with substance misuse. Increased staffing, such as extended hour ED-based psychiatric nurse practitioners [30], and the development of dedicated psychiatric assessment units supported by ED and staffed with a full multidisciplinary team have shown benefit [31], however are resource heavy. Current data remains limited regarding completion of care, with an emphasis on reducing repeat presentations.

Further qualitative data is required to understand the needs of this population and how incomplete care can be avoided in those often hardest to reach. Given the reports of significantly elevated rates of suicide in the population attending the ED for self-harm, departure before completion of care is a substantial safety concern [12–14]. Addressing the factors which create barriers to care will improve the patient experience overall, while reducing disruption and significant risk associated with leaving before completion of care.

## Limitations

In this study, those with mental health presentations to the ED were identified via the attendance of registers, relying on initial triage codes. In this approach, there were likely a significant cohort presenting with mental health indications triaged as physical complaints, such as trauma, shortness of breath, or other vague self-reported symptoms. This may have resulted in the under-reporting of the true number of mental health presentations.

In this dataset, it was not possible to ascertain whether ambulance transfer was initiated by the patient themselves, or a third party. Details regarding the reason for leaving the ED, or where along the treatment pathway they were when leaving were also not available. Further qualitative data would provide additional information into the characteristics of those leaving ED prior to completion of care.

## Conclusions

This study outlines the characteristics of those attending the ED with mental health indications, and any association with self-discharge prior to completion of care. In the subgroup of people attending the ED with mental health problems, self-discharge is associated with those transferred by ambulance, triaged as ‘overdose and poisoning’, male gender, and those who present in the late evening/night. These patients may need to be prioritised and target interventions employed to reduce their rates of incomplete care. Further qualitative data is required to understand the needs of this population and the factors influencing the premature cessation of treatment. Parallel assessment, where medical investigations and psychiatric evaluation occur in tandem, has the potential to reduce wait times and increase the overall success of emergency intervention.

## Data Availability

The datasets analysed during the current study are not publicly available due to the data protection policies of the institution, but are available from the corresponding author on reasonable request.

## Abbreviations

ED: Emergency Department
EPR: Electronic Patient Records
